# Characterisation of the global transcriptional response to heat shock and the impact of individual genetic variation

**DOI:** 10.1186/s13073-016-0345-5

**Published:** 2016-08-24

**Authors:** Peter Humburg, Narelle Maugeri, Wanseon Lee, Bert Mohr, Julian C. Knight

**Affiliations:** 1 Wellcome Trust Centre for Human Genetics, University of Oxford, Oxford, UK; 2 Queensland Institute of Medical Research, Brisbane, 4029 Queensland Australia; 3 Hatter Institute for Cardiovascular Research in Africa, Department of Medicine, Faculty of Health Sciences, University of Cape Town, Cape Town, South Africa

## Abstract

**Background:**

The heat shock transcriptional response is essential to effective cellular function under stress. This is a highly heritable trait but the nature and extent of inter-individual variation in heat shock response remains unresolved.

**Methods:**

We determined global transcription profiles of the heat shock response for a panel of lymphoblastoid cell lines established from 60 founder individuals in the Yoruba HapMap population. We explore the observed differentially expressed gene sets following heat shock, establishing functional annotations, underlying networks and nodal genes involving heat shock factor 1 recruitment. We define a multivariate phenotype for the global transcriptional response to heat shock using partial least squares regression and map this quantitative trait to associated genetic variation in search of the major genomic modulators.

**Results:**

A comprehensive dataset of differentially expressed genes following heat shock in humans is presented. We identify nodal genes downstream of heat shock factor 1 in this gene set, notably involving ubiquitin C and small ubiquitin-like modifiers together with transcription factors. We dissect a multivariate phenotype for the global heat shock response which reveals distinct clustering of individuals in terms of variance of the heat shock response and involves differential expression of genes involved in DNA replication and cell division in some individuals. We find evidence of genetic associations for this multivariate response phenotype that involves trans effects modulating expression of genes following heat shock, including *HSF1* and *UBQLN1*.

**Conclusion:**

This study defines gene expression following heat shock for a cohort of individuals, establishing insights into the biology of the heat shock response and hypotheses for how variation in this may be modulated by underlying genetic diversity.

**Electronic supplementary material:**

The online version of this article (doi:10.1186/s13073-016-0345-5) contains supplementary material, which is available to authorized users.

## Background

The heat shock response is a highly conserved mechanism found across organisms that ensures effective maintenance of cellular function under stress. Transcriptional activation involving heat shock proteins (HSPs) was found to underpin the seminal observation of expanded chromosomal puffs in *Drosophila* salivary glands following exposure to heat [1], with subsequent studies in different species highlighting not only changes in expression of genes encoding these essential molecular chaperones but also their regulators, proteins involved in proteolysis, transcription factors and kinases, membrane transport, maintenance of cellular structures, metabolism and nucleic acid repair [2–9]. As well as significant upregulation of gene expression, involving rapid induction of HSP gene transcription by activated heat shock factors (HSF) binding to promoter heat shock elements (HSEs), the coordinated stress response is also recognised to involve downregulation of a greater number of genes. However, to date inter-individual variation in the heat shock response at the level of transcription in humans remains largely unknown, with studies defining the global transcriptome based on specific cell lines or cells/tissue from particular individuals [8, 9]. Further delineation of the nature and variability in this response is important given the role of HSPs in ensuring effective intracellular protein folding during stress, protecting cells from denaturation, aggregation and apoptosis [4]. This is underlined by evidence linking HSPs with ageing and cancer, as well as the response to infection and immunity [10–13].

Genetic modulators of gene expression are important determinants of inter-individual variation in diverse phenotypes and may only operate in specific cell types or after particular environmental exposures [14, 15]. Mapping gene expression as a quantitative trait to identify regulatory genetic variants has informed recent genome-wide association studies (GWAS) of disease as well as pathophysiology including the immune response to endotoxin [16], sepsis [17], T-cell activation [18] or viral infection [19, 20]. Expression of heat shock proteins is highly heritable and has been mapped as a quantitative trait in diverse organisms including *Drosophila melanogaster* [21–23], *Caenorhabditis elegans* [24] and the Artic charr [25]. In resting (non-heat shocked) human Epstein-Barr virus (EBV)-immortalised lymphoblastoid cell lines (LCLs), expression of heat shock protein and molecular chaperone genes shows high heritability on eQTL mapping, with response to unfolded proteins having the highest heritability of any biological process on gene ontology (GO) analysis (H^2^ 0.38) [26]. A previous QTL analysis of heat shock phenotypes in human cells was restricted to the Hsp70 genes in the MHC class II region and demonstrated a local eQTL for *HSPA1B* [27].

Here we report the genome-wide changes to gene expression induced by heat shock in HapMap cell lines from Yoruba (YRI) individuals and perform analysis to identify genes and pathways involved in the human heat shock response. To further elucidate underlying mechanisms, we present an analysis of genetic variants modulating the global heat shock transcriptional response.

## Methods

### Cell culture and heat shock

The 60 founder YRI HapMap cell lines (Coriell) [28] were cultured. These anonymised cell lines were established by the International HapMap Project and made available for use by the scientific research community [29]. LCLs were maintained in RPMI 1640 medium supplemented with 10 % fetal calf serum and 2 mM L-glutamine at 37 °C in 5 % humidified CO_2_. Growth rates were determined after 72 h in culture for each cell line to ensure the cells were at comparable densities and total numbers when harvested. Trypan blue staining was used to define cell viability. Cells were subject to heat shock at 42 °C for 1 h and then allowed to recover for 6 h in a 37 °C, 5 % CO2 incubator. 2 × 10^7^ cells were harvested for each of the two paired experimental conditions (i.e. heat shock stimulated and basal un-stimulated culture conditions) per individual cell line and stored in RLT buffer with β-mercaptoethanol at −80 °C. Total RNA was purified using QIAGEN RNeasy Mini purification kit following manufacturer’s instructions, including on-column DNase digestion.

### Gene expression pre-processing and quality control

Genome-wide gene expression analysis was carried out using the Illumina Human-HT-12 v3 Expression BeadChip gene expression platform comprising 48,804 probes. Probe intensities for resting and stimulated cells were imported into R for further processing together with associated metadata. Annotations for all probes were obtained via the illuminaHumanv3.db Bioconductor package [30]. Only probes considered to be of perfect or good quality according to these annotations were taken forward for analysis. Additionally, all probes mapping to more than one genomic location or to a location that contains a known single nucleotide polymorphism (SNP) were excluded. Probes were required to exhibit significant signal (detection *p* value <0.01) in at least ten samples and samples with less than 30 % of the remaining probes providing significant signal were excluded (together with the paired sample from the same cell line). Samples showing exceptionally low variation in probe intensities (standard deviation of the log intensities of all retained probes below 0.8) were also removed. After filtering 12,416 of 48,803 probes (25.4 %) remained.

### Normalising gene expression estimates

Probe intensities were normalised with VSN [31] and outlier samples removed. The remaining 43 samples were normalised separately for each BeadChip and differences between groups corrected with ComBat [32], preserving differences due to heat shock stimulation (Additional file 1: Figure S1).

### Differential expression analysis

Following quality control (QC), samples were analysed for differences in gene expression levels between the basal and stimulated states, i.e. pairing samples from the same individual, using the limma Bioconductor package [33]. Individual probes were associated with corresponding genes by comparing probe positions as provided by the illuminaHumanv3.db Bioconductor package [30] with transcript coordinates obtained via the TxDb.Hsapiens.UCSC.hg19.knownGene Bioconductor package [34]. One of the genes (*N4BP2L2*) had two probes with opposite effects in terms of differential expression and these probes were excluded from further analysis. For all other genes with multiple differentially expressed probes, the direction of the effect was consistent between probes.

### GO enrichment and pathway analysis

GO enrichment analysis was carried out using the Bioconductor package topGO [35]. Fisher’s exact test was used to determine enrichment separately for significantly upregulated and downregulated genes (false discovery rate (FDR) <0.01 and >1.2 fold change (FC)). Biological pathways, function enrichment and prediction of upstream regulators were generated for these genes using Qiagen’s Ingenuity Pathway Analysis (IPA) (www.qiagen.com/ingenuity, QIAGEN Redwood City). For the shortest path analysis, we used the path explorer tool. Here, if two molecules do not have specific direct connections in the Ingenuity Knowledge Base, this tool will define how many and which molecules can be added to the pathway to create the shortest path between them.

### Gene functional annotations with heat shock

We investigated which differentially expressed genes we identified had been previously associated with the heat shock or, more generally, stress response. We used the set of genes previously linked directly to heat shock [4] and from this created an extended set based on GO terms and PubMed articles linking differentially expressed genes to heat shock response and closely related processes. As a first step in highlighting genes not previously known to play a role in this context, we identified all significantly upregulated genes that lack GO annotations of obvious relevance to heat shock response. In addition to terms related to stress response and protein folding, we also explored an extended set that included terms related to cell death and proliferation. To account for the presence of EBV in these cell lines, we excluded all genes annotated with terms related to viral infections. Finally, any remaining genes related to regulation of gene expression were considered to be likely to be explained by the large-scale changes in gene expression that are taking place in response to heat shock and also included in the extended set. All genes not annotated with obvious GO terms were subjected to a PubMed search to find publications that link the gene to heat shock or stress response.

### Heat shock factor binding

Using binding sites derived from ChIP-seq data obtained from the K562 immortalised leukaemic cell line [36], we annotated our list of differentially expressed genes by cross-referencing it with the list of HSF-binding genes. Groups of genes corresponding to upregulated or downregulated genes as well as those with existing heat shock-related annotations and those without were tested for enrichment of HSF-binding genes using Fisher’s exact test. In addition to the direct evidence from the ChIP-seq data, we carried out a scan for the presence of HSF-binding motifs in the promoter region (1200 bp upstream–300 bp downstream of the transcriptional start site (TSS)) of differentially expressed genes. The scan was based on the position weight matrices (PWM) defined by SwissRegulon [37] and carried out with the Bioconductor package PWMEnrich [38].

### Multivariate global heat shock response phenotype

The global heat shock response was summarised using partial least squares (PLS) regression (generated as detailed in ‘Results’). Using the first two PLS components with respect to the treatment, i.e. the two components of the gene expression space that maximise the variation between basal and stimulated samples, we defined the response for each individual as the combination of the vector between the basal and stimulated sample for this individual in the space spanned by the first two PLS components and the location of the basal sample in the same space. Hierarchical cluster analysis was used to investigate grouping of individuals following heat shock and differential gene expression between clusters analysed.

### Genotype QC

Genotype data provided by the HapMap project [39] were processed with Plink [40] to restrict the data to autosomes and remove SNPs with low genotyping rate and those with a minor allele frequency of less than 10 % in our sample set. This resulted in the exclusion of 794,511 of 2,582,999 SNPs (30.76 %). Estimation of the proportion of identity by descent for all sample pairs demonstrated three pairs showing evidence of higher than expected relatedness (Additional file 2: Figure S2) which was supported by IBS nearest neighbour calculation. As a result, samples NA18913, NA19192, NA18862 and NA19092 were excluded.

### Genotypic association with gene expression

The multivariate global heat shock response phenotype was tested for association with SNPs within a 10 kb window either side of the probe location using the MultiPhen R package [41], 10 kb selected as informative for including functional elements interacting with a gene [42, 43]. All differentially expressed probes and all probes involving predicted upstream regulator genes were analysed but only genotyped SNPs that passed QC were considered. The GRCh37 coordinates for SNPs were obtained via the SNPlocs.Hsapiens.dbSNP142.GRCh37 Bioconductor package [44] and gene coordinates via the TxDb.Hsapiens.UCSC.hg19.knownGene package [34]. The significance of the observed associations was assessed through a permutation test to account for the structure inherent to the data. To this end the observed global response phenotype for each individual and covariates used in the model were randomly assigned to one of the observed set of genotypes 1000 times and *p* values for the joint model were computed for each permutation. From these we computed FDRs by comparing observed *p* values to the empirical distribution of minimum *p* values from each permutation. We tested for associations between genotype and heat shock response (log_2_ FC) for individual genes using a linear model as implemented in Matrix-eQTL [45], correcting for sex as well as the first two principal components of the treatment response to capture confounding variation, an approach which enhances eQTL mapping [46–48].

## Results

### Transcriptomic response to heat shock

We aimed to establish the nature and extent of inter-individual variation in the genome-wide transcriptomic response to heat shock for a panel of LCLs established from unrelated individuals of African ancestry for whom high-resolution genotyping data are available (International HapMap Project, YRI population) [28]. We cultured the LCLs and exposed cells to heat shock at 42 °C for 1 h and harvested after recovery at 37 °C for 6 h. We then quantified genome-wide gene expression using Human-HT-12 v3 Expression BeadChips (Illumina). Following QC and processing, paired expression data (baseline and following heat shock) were available for 12,416 probes on 43 individual cell lines.

**Fig. 1:**
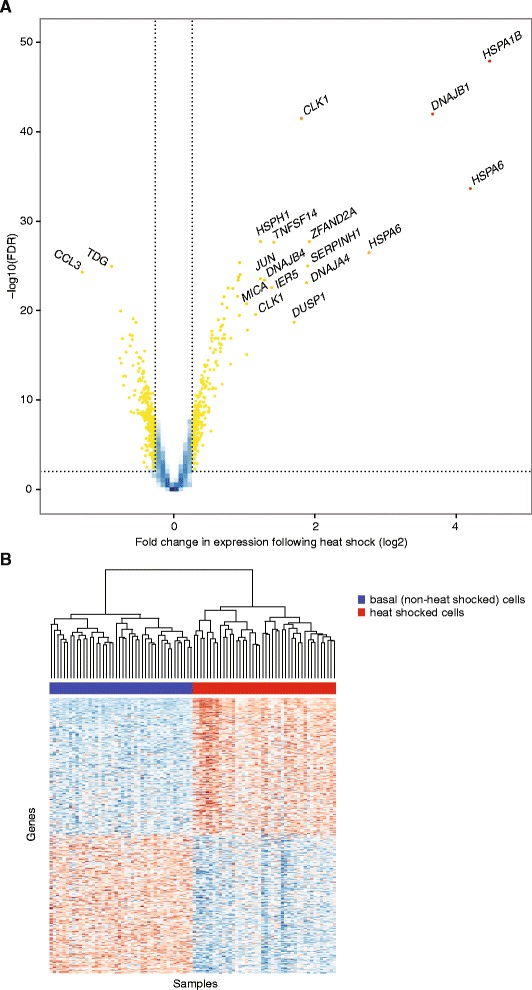
Heat shock response in LCLs. **a** *Volcano plot* showing differentially expressed genes following heat shock (42 °C for 1 h with 6 h recovery) in LCLs. Probes with an adjusted *p* value below 0.01 and a log FC of at least 0.5 are shown as *yellow* and *red dots*. Probes showing particularly strong evidence of changes in gene expression through a combination of *p* value and FC are labelled with the corresponding gene symbol. **b** *Heatmap* comparing gene expression for differentially expressed genes between basal and stimulated samples. Samples were clustered by gene with heat shocked (*red*) and basal (*blue*) samples forming two distinct groups. Expression estimates for each gene were scaled and centred across samples. *Blue cells* correspond to lower than average expression and *red cells* correspond to higher than average expression

We found that 500 probes (4 % of all analysed probes, corresponding to 465 genes) were differentially expressed (FDR <0.01 and >1.2 FC) with 249 probes (226 genes) upregulated and 251 probes (238 genes) downregulated (Fig. 1, Table 1, Additional file 3: Table S1). The majority of the most significantly differentially expressed probes were upregulated, including 18 of the top 20 genes, of which nine encoded known heat shock proteins. The most significant difference in expression was seen for *HSPA1B* (22.2 FC, FDR 1.4 × 10^−48^).

**Table 1:** Top 20 differentially expressed genes following heat shock

Gene	EntrezID	logFC	FC	Average expression	t	*p* value	Adjusted *p* value	B
*HSPA1B*	3304	4.5	22.2	11.9	53.9	1.1E-52	1.4E-48	105.5
*DNAJB1*	3337	3.7	12.7	10.9	42.2	1.8E-46	1.1E-42	93.4
*CLK1*	1195	1.8	3.5	10.8	41.1	8.4E-46	3.5E-42	92.0
*HSPA6*	3310	4.2	18.3	9.4	29.9	7.8E-38	2.4E-34	75.2
*HSPH1*	10808	1.2	2.3	12.7	23.3	8.2E-32	2.0E-28	61.9
*ZFAND2A*	90637	1.9	3.8	9.5	23.2	9.8E-32	2.0E-28	61.8
*TNFSF14*	8740	1.4	2.7	9.4	23.0	1.4E-31	2.5E-28	61.4
*HSPA6*	3310	2.8	6.8	7.8	21.9	2.3E-30	3.6E-27	58.7
*FXR1*	8087	0.9	1.9	9.9	20.8	3.3E-29	4.5E-26	56.1
*SERPINH1*	871	1.9	3.7	8.7	20.4	8.6E-29	1.1E-25	55.2
*TDG*	6996	−0.9	0.5	10.7	−20.4	1.1E-28	1.2E-25	55.0
*CCL3*	6348	−1.3	0.4	11.4	−19.8	5.1E-28	5.2E-25	53.4
*KIAA0907*	22889	0.9	1.9	9.5	19.5	1.0E-27	9.9E-25	52.7
*HSPA4L*	22824	0.9	1.9	9.3	19.2	2.2E-27	2.0E-24	52.0
*JUN*	3725	1.2	2.3	9.2	19.0	3.5E-27	2.9E-24	51.5
*CACYBP*	27101	0.9	1.9	11.4	18.9	5.7E-27	4.2E-24	51.1
*DNAJB4*	11080	1.3	2.4	7.2	18.9	5.8E-27	4.2E-24	51.0
*IER5*	51278	1.4	2.6	10.5	18.1	4.3E-26	2.8E-23	49.1
*LMAN2L*	81562	0.8	1.7	8.4	18.1	4.9E-26	3.1E-23	48.9
*BANP*	54971	0.8	1.8	10.8	18.0	5.9E-26	3.47E-23	48.8

The most significant differentially expressed genes for a panel of LCLs exposed to heat shock (42 °C for 1 h, 6 h recovery) and assayed by microarray are shown following limma analysis

To further investigate the patterns of transcriptional response, we carried out a GO enrichment analysis for differentially expressed genes (>1.2 FC, FDR <0.01). This demonstrated significant enrichment among upregulated genes (seven categories with an FDR <0.05 on Fisher’s exact test) but no significant enrichment for downregulated genes (Table 2, Additional file 3: Tables S2 and S3). Considering the top categories, we found that genes upregulated following heat shock were predominantly related to the response to heat (including GO:0009408) and to unfolded protein (GO:0006986), together with negative regulation of inclusion body assembly (GO:0090084), endoplasmic reticulum stress (GO:1903573) and cell death (GO:0060548).

**Table 2:** GO categories enriched for upregulated and downregulated genes

GO ID	Term	Annotated genes	Significant	Expected	Rank in downregulated genes	*p* value (FDR) for upregulated genes	*p* value (FDR) for downregulated genes
(A) Top GO categories enriched for upregulated genes							
GO:0009408	Response to heat	70	13	1.51	2483	5.7 × 10^−8^ (2.3 × 10^−4^)	0.87 (1)
GO:0006986	Response to unfolded protein	97	15	2.09	2757	7.1 × 10^−8^ (2.7 × 10^−4^)	1 (1)
GO:0006457	Protein folding	133	17	2.87	1600	1.7 × 10^−7^ (6.4 × 10^−4^)	0.52 (1)
GO:0035966	Response to topologically incorrect protein	106	15	2.29	2368	2.4 × 10^−7^ (9.4 × 10^−4^)	0.81 (1)
GO:0009266	Response to temperature stimulus	96	13	2.07	2257	2.5 × 10^−6^ (9.8 × 10^−3^)	0.76 (1)
GO:0042026	Protein refolding	11	5	0.24	2758	6.7 × 10^−6^ (0.028)	1 (1)
GO:0034605	Cellular response to heat	50	9	1.08	2759	8.6 × 10^−6^ (0.035)	1 (1)
GO:0043618	Regulation of transcription from RNA polymerase II promoter in response to stress	35	7	0.76	1907	4.3 × 10^−5^ (0.18)	0.63 (1)
GO:1900034	Regulation of cellular response to heat	26	6	0.56	2760	6.6 × 10^−5^ (0.27)	1 (1)
GO:0043620	Regulation of DNA-templated transcription in response to stress	39	7	0.84	2008	9 × 10^−5^ (0.37)	0.67 (1)
(B) Top GO categories enriched for down regulated genes							
GO:0051225	Spindle assembly	37	6	0.82	1	1 (1)	5.4 × 10^−4^ (1)
GO:0043207	Response to external biotic stimulus	342	20	7.59	2	0.63 (1)	1.6 × 10^−3^ (1)
GO:0051707	Response to other organism	342	20	7.59	3	0.63 (1)	1.6 × 10^−3^ (1)
GO:0045931	Positive regulation of mitotic cell cycle	64	7	1.42	4	1 (1)	2.1 × 10^−3^ (1)
GO:0007049	Cell cycle	1037	45	23.02	5	0.69 (1)	2.2 × 10^−3^ (1)
GO:0007143	Female meiotic division	10	3	0.22	6	1 (1)	2.3 × 10^−3^ (1)
GO:0009607	Response to biotic stimulus	355	20	7.88	7	0.54 (1)	2.6 × 10^−3^ (1)
GO:0032496	Response to lipopolysaccharide	128	10	2.84	8	0.49 (1)	3.3 × 10^−3^ (1)
GO:1903047	Mitotic cell cycle process	552	27	12.26	9	0.78 (1)	3.7 × 10^−3^ (1)
GO:0002237	Response to molecule of bacterial origin	134	10	2.98	10	0.52 (1)	4.6 × 10^−3^ (1)
GO:0008219	Cell death	1022	43	22.69	11	4.3 × 10^−3^ (1)	4.9 × 10^−3^ (1)

The most significant GO categories for differentially expressed genes following heat shock in LCLs are shown. Numbers of significant and expected genes shown, together with *p* values (Fisher’s exact test)

We then performed pathway analysis of differentially expressed genes. Using IPA we found that the most significantly enriched canonical pathway among upregulated and downregulated genes (>1.2 FC, FDR <0.01) was the unfolded protein response (*p* value 6.8 × 10^−8^). We also found that heat shock factor 1 (HSF1) was the most significant upstream regulator (*p* value 2.5 × 10^−13^). Further investigation established that 81 % of observed differentially expressed genes were linked to HSF1 directly or through one additional molecule based on shortest path analysis using the Ingenuity Knowledge Base (Additional file 4: Figure S3). In addition to networks involving heat shock protein genes, this analysis highlighted the role of ubiquitination (UBC) and sumoylation (SUMO2, SUMO3) as well as transcription factors (including NFkB, JUN, ATF2, CEBP) and cytokines (IL6 and TNF) in the observed heat shock response at the transcriptional level (Additional file 4: Figure S3). In terms of biological functions, we resolved using IPA that cell death (*p* value 2.2 × 10^−8^), cell proliferation (*p* value 3.6 × 10^−8^), apoptosis (*p* value 8.2 × 10^−8^), cell cycle (*p* value 2.6 × 10^−7^) and gene expression (*p* value 6.6 × 10^−7^) were most significantly enriched. Upregulated and downregulated genes were found to cluster in a number of highly enriched networks constructed from the Ingenuity Knowledge Base (Additional file 3: Table S4).

### Heat shock factor recruitment

Of the 226 significantly upregulated genes following heat shock, 24 genes have been previously directly linked to the heat shock response. We found that there was significant enrichment for genes associated with GO terms that clearly relate to heat shock response with 98 genes annotated with such terms (*p* value 2.3 × 10^−10^, Fisher’s exact test) and 21 otherwise linked to the heat shock response as revealed by a text mining strategy (detailed in ‘Methods’). Additionally, 30 genes were annotated with other relevant processes. This leaves 53 genes with no obvious previous association to heat shock.

To further establish links between differentially expressed genes and heat shock response, we considered the evidence for binding of HSF1 and HSF2 in the promoter regions of upregulated genes using ChIP-seq data obtained for K562 cells following heat shock [36]. Overall there was significant enrichment of HSF1 (51 genes, *p* 4.7 × 10^−10^ on Fisher’s exact test, odds ratio (OR) 3.0), HSF2 (55 genes, *p* 9.4 × 10^−9^, OR 2.6) and binding of both HSF1 and HSF2 (46 genes, *p* 9.1 × 10^−15^, OR 4.5) among upregulated genes following heat shock. Of the nine upregulated genes following heat shock without an established role where we find evidence of HSF binding on ChIP-seq (Additional file 3: Table S5), four have HSF-binding motifs in the promoter region (Additional file 3: Table S6).

### Variation in the global heat shock response

**Fig. 2:**
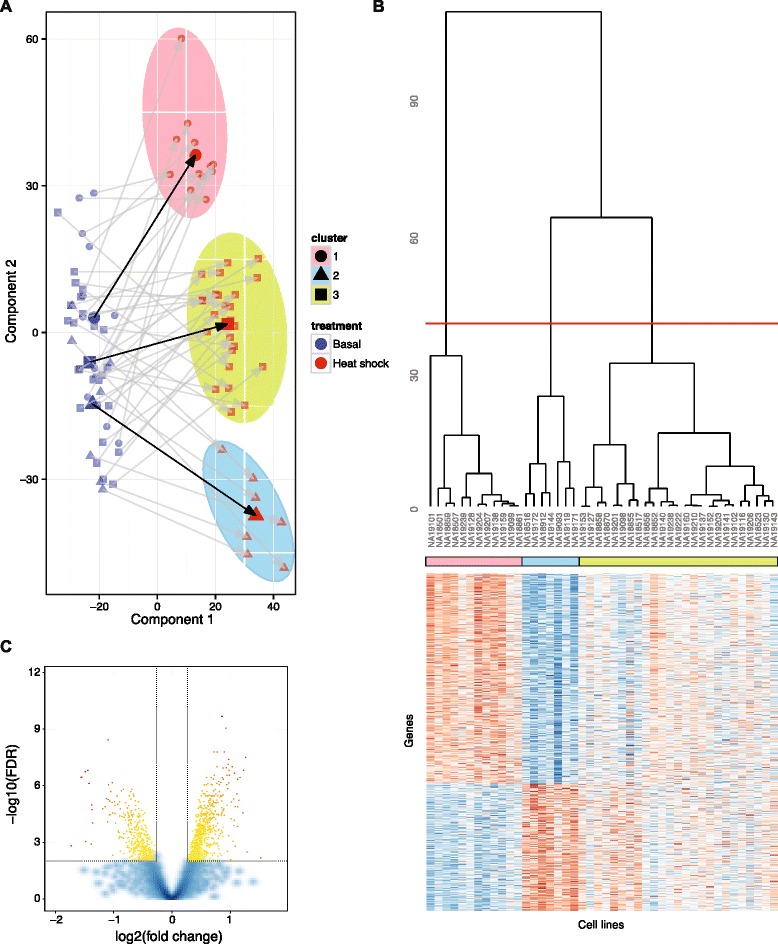
Variance in the global heat shock response. **a** Modelling of the genome-wide transcriptional response to heat-shock (component plot) based on PLS to identify latent structures in the data for cohort of 43 LCLs. The *x-axis* represents the first PLS component which segregates basal samples (*left*) and heat shocked samples (*right*). The *y-axis* represents the second PLS component which involves variation between cell lines in basal and heat shock response states. Each cell line’s basal and heat shock samples are similarly coloured and paired samples are connected with an arrow, which represents the vector used as quantitative trait in the genetic association test for genetic modulators of the global heat shock response. The average response is indicated by a *black arrow*. Overall, samples separate clearly by treatment, showing a consistent global effect on gene expression from heat shock. Heat shock stimulated samples show evidence of three distinct clusters (indicated by *shaded ovals*). **b** Unsupervised hierarchical cluster analysis with heat shock stimulated samples showing evidence of three distinct clusters (indicated on panel A by *shaded ovals*). Below the cluster dendrogram is a *heatmap* showing differential gene expression. Expression estimates for each gene were scaled and centred across samples. *Blue cells* correspond to lower than average expression and *red cells* correspond to higher than average expression. **c** *Volcano plot* of differential expression results between clusters 1 and 2. Probes with an adjusted *p* value below 0.01 and a log FC of at least 0.5 are shown as *yellow* and *red dots*

To assess the global difference in gene expression induced by heat shock, we carried out PLS, using the treatment state (basal or following heat shock) as a binary response variable and all gene expression probes that passed QC as explanatory variables (12,416 probes targeting 10,214 genes). PLS has been previously used to identify differentially expressed genes [49] and coordinated expression profiles [50] including global response phenotypes [51]. The supervised PLS approach identifies variance components that differentiate treatment groups. This contrasts with principal component analysis (PCA), which considers overall variance irrespective of any known groupings. The PLS analysis demonstrated that there is a considerable change in overall gene expression in response to heat shock with the first two PLS components together accounting for 96.1 % of the variation observed and providing clear separation of the two treatment groups (Fig. 2).

In addition to the pronounced shared response to heat shock that is largely accounted for by the first component, a further effect related to differences in the individual response is noticeable in the second component. This manifests in a visually striking grouping of samples into three clusters post treatment (Fig. 2). To further characterise the difference between these clusters we carried out a differential expression analysis between the two clusters that differ most with respect to the second PLS component. Using an FDR threshold of 0.01 and requiring a FC of at least 1.2, this identified 1094 differentially expressed probes (Additional file 3: Table S7). Of these 681 are upregulated and 415 are downregulated in cluster 2 compared to cluster 1 (Fig. 2).

To further investigate which biological processes underlie the observed differences, we carried out a GO analysis of genes exhibiting significantly increased expression in either cluster. GO categories enriched in the set of genes upregulated in cluster 2 are largely similar to those identified in the analysis of genes that show increased expression in response to heat shock, including response to unfolded protein (GO:0006986) and response to topologically incorrect protein (GO:0035966) (Additional file 3: Table S8). In contrast, genes with higher expression in cluster 1 are enriched for GO annotations relating to DNA replication and cell division including DNA recombination (GO:0006310) and DNA replication (GO:0006260) (Additional file 3: Table S9).

**Fig. 3:**
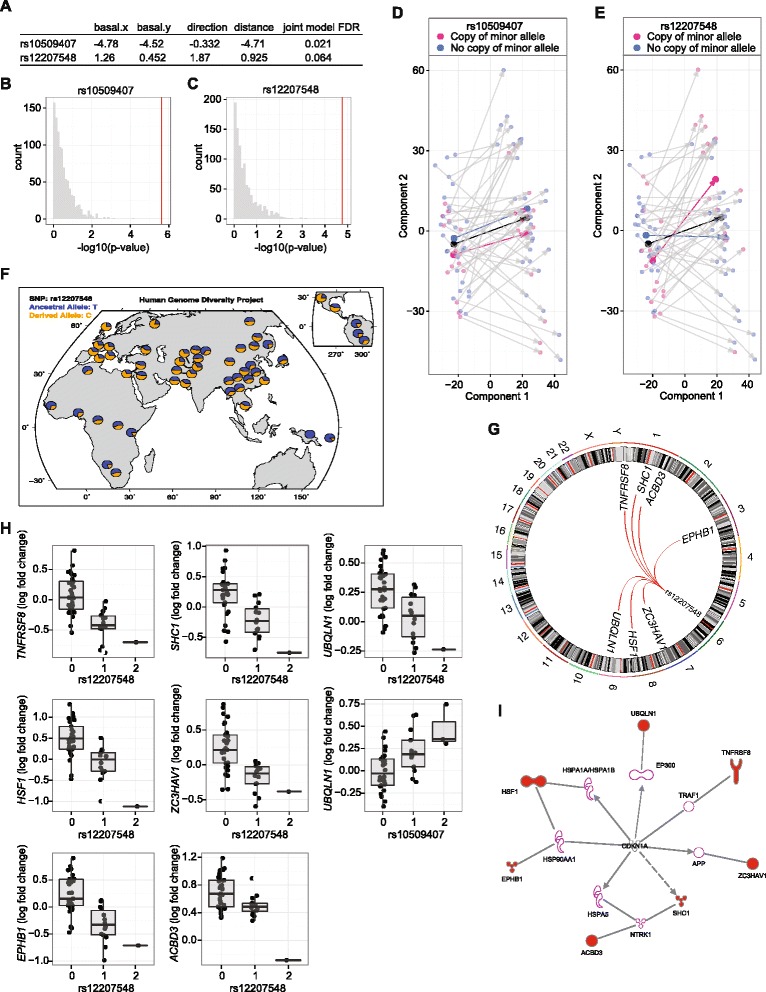
Genotypic association with global heat shock response. **a** Standardized coefficients and adjusted *p* values for the top associated SNPs. **b**, **c** The distribution of *p* values after permutation of the global response phenotype is shown for rs10509407 (**b**) and rs12207548 (**c**). **d**, **e** Global response to heat shock showing individual LCLs by genotype for rs10509407 (**d**) and rs12207548 (**e**). Each individual is represented by two points corresponding to basal and stimulated state with *arrows* connecting paired samples. Genotypes are indicated by colour with *blue* corresponding to homozygous carriers of the major allele and *red* indicating the presence of at least one copy of the minor allele. *Coloured arrows* show the average response for each group. The overall average is indicated in *black*. **f** Ancestral Allele Frequencies for rs12207548 from Human Genome Diversity Project in 53 populations. **g** *Circos plot* showing trans associations for rs12207548. **h** *Box plots* for expression of *UBQLN1*, *HSF1*, *TNFRSF8*, *EPHB1*, *SHC1*, *ZC3HAV1* and *ABCD3* by allele for SNPs as indicated. **i** *Pathway analysis* using IPA showing links between trans associated genes for rs12207548 and CDKN1A

To explore to what extent this response is modulated by genetic variation, we used the length and direction of the response vector, i.e. the vector between the basal and stimulated sample for each individual in the space spanned by the first two PLS components, together with the location of the basal sample in the same space, as a multivariate phenotype. This was then tested for association with genotypes for SNPs within a 10-kb window of differentially expressed genes following heat shock or genes encoding predicted upstream regulators of differentially expressed genes identified by IPA analysis. This revealed two significant associations (Fig. 3). The first involved rs10509407 (FDR 0.021), a promoter variant of *MINPP1* (encoding endoplasmic reticulum luminal enzyme multiple inositol polyphosphate phosphatase), which was in complete linkage disequilibrium with three further SNPs. The other association we identified involved rs12207548 (FDR 0.064), a regulatory variant located in a CTCF binding site 1.14 kb downstream of *CDKN1A. CDKN1A* is an important regulator of cell cycle progression. The SNP rs12207548 shows significant variation in allele frequency between human populations (Fig. 3) with an estimated F_ST_ of 0.142 (the F_ST_ providing a summary of the genetic differentiation between these populations).

To explore the observed association between heat shock response and genotypes at these two loci, we proceeded to test for association with differential expression (FC) following heat shock for individual genes with the two identified variants. We found evidence that both SNPs show trans association with differential induction of *UBQLN1* after heat shock (rs10509407 FDR 0.011, beta 0.232; rs12207548 FDR 0.010, beta –0.238) (Fig. 3). *UBQLN1* encodes ubiquilin, which is involved in protein degradation by linking the ubiquitination machinery to the proteasome. We found that rs12207548 was also associated with a trans network involving differential expression of six further genes: *HSF1* (FDR 0.00075, beta –0.643); *TNFRSF8* (FDR 0.00075, beta –0.477); *EPHB1* (FDR 0.00075, beta –0.532); *SHC1* (FDR 0.0031, beta –0.456); *ZC3HAV1* (FDR 0.0036, beta –0.399) and *ABCD3* (FDR 0.010, beta –0.279) (Fig. 3). Network analysis using IPA highlights the relationship of these trans genes, either directly or involving additional molecules, with CDKN1A (Fig. 3).

## Discussion

We have generated a comprehensive catalogue of differential gene transcription following heat shock for human LCLs, significantly expanding the number of genes recognised to be upregulated and downregulated by exposure of cells to heat shock [4, 8, 9]. We have shown how this relates to HSF1 and HSF2 recruitment and determined several key nodal molecules in the observed pattern of differential expression using a network approach. This includes a role for ubiquitin C and small ubiquitin-like modifiers SUMO2/3 as well as heat shock proteins, transcription factors (NFkB, CEBP, JUN) and cytokines (TNF, IL6). Given that transcriptomic differences may not be reflected at a protein level [52], complementary proteomic analysis such as used to define stress-independent HSF1 activation in a ligand-mediated cell line model system would be informative [53].

We have investigated variation in the global heat shock response across individual LCLs, defining a multivariate phenotype using PLS which revealed evidence of clustering with relative predominance of differential expression of genes involved in DNA replication and cell division in some individuals. We further investigated specific genotypic associations with the observed variation which revealed associations with putative regulatory variants, tagged by rs10509407 and rs12207548 located in/near the genes *MINPP1* and *CDKN1A*, key genes involved in cell growth and survival. These SNPs show trans association with differential expression following heat shock of *UBQLN1* (ubiquilin), an important mediator of degradation of proteins in the stress response [54] implicated in Alzheimer’s disease [55], and a network of six further genes including *HSF1*. However, we did not observe cis-associations with expression of *MINPP1* and *CDKN1A* which leaves unresolved the cis-drivers of the observed trans associations. This may require additional time points of sampling to capture such cis-effects, as illustrated by our recent studies of trans-eQTL following endotoxin induction [16].

Our results are necessarily exploratory given the modest sample size of this study requiring further validation and functional characterisation to establish mechanism. If functionally validated, the geographic distribution of the major and minor alleles of rs12207548 suggests selection may be operating on such variants. We recognise that there may be cell type-specific differences in heat shock response not captured by our analysis in LCLs, including differences in HSF binding from the K562 cell line, and that there may also be population specific differences in terms of regulatory variants with the data presented here generated in cells from individuals of African ancestry. We elected to follow a focused high-level approach in this paper as we are not adequately powered for a systematic QTL analysis of all individual genes.

Our approach to analysing the global transcriptional response to stimuli or treatment as a multivariate phenotype provides a single global phenotype for analysis, rather than several thousands of gene-level phenotypes, which is more robust to probe-level technical artefacts and reduces the number of multiple comparisons as well as computational cost of eQTL analysis, especially for omics-scale data. We suggest it is broadly applicable and relevant to other phenotypes in which modulation by genetic variation may be sought. These are highlighted by recent work that has demonstrated the context-specificity of regulatory variants including different disease contexts through QTL approaches in patient samples [15]. For the inflammatory response, these can be complemented by analysis ex vivo of specific phenotypes such as heat shock.

## Conclusions

We have defined the global transcriptional response to heat shock for a panel of human B lymphocyte cell lines, establishing a comprehensive catalogue of differentially expressed genes, pathways and networks of broad utility to understand this highly conserved and pathophysiologically significant response. We have also explored the genetic basis for inter-individual variation in the global response, highlighting putative regulatory variants modulating ubiquilin and a further trans gene network.

## Additional files

Additional file 1: Figure S1.
*PCA plot* of ComBat corrected gene expression. *PCA plot* for gene expression in LCLs following heat shock post microarray processing and QC with individual lines coloured by BeadChIP. (PDF 166 kb) Additional file 2: Figure S2.
*Dendrogram* of individuals included in study. *Plot* showing distances based on identity by state for LCLs from HapMap (YRI) included in this study. Three pairs show clear indications of increased relatedness. (PDF 131 kb) Additional file 3: Table S1. Differentially expressed genes following heat shock. Differentially expressed genes for a panel of 43 LCLs exposed to heat shock (42 °C for 1 h, 6 h recovery) and assayed by microarray are shown following limma analysis (FC >1.2, FDR <0.01). **Table S2.** GO categories enriched for upregulated genes. GO categories for differentially expressed genes upregulated following heat shock in LCLs are shown. Numbers of significant and expected genes shown, together with *p* values (Fisher’s exact test). **Table S3.** GO categories enriched for downregulated genes. GO categories for differentially expressed genes downregulated following heat shock in LCLs are shown. Numbers of significant and expected genes shown, together with *p* values (Fisher’s exact test). **Table S4.** Network analysis following heat shock. Networks identified on IPA analysis of differentially expressed genes (FC >1.2, FDR <0.01) following heat shock. **Table S5.** Genes with newly established links to heat shock response. Genes listed together with FC and FDR following heat shock, and *p* value for presence of the heat shock binding motif. **Table S6.** Summary of HSF-binding evidence for the promoters of novel and established heat shock response genes. Presence of ChIP-seq peak for HSF1 or HSF2 and HSF1 motif indicated in relation to heat shock genes. **Table S7.** Differential gene expression between PLS clusters. Differential gene expression between samples assigned to PLS cluster 1 and 2 as assessed by limma analysis is shown for all assayed probes. **Table S8.** GO categories enriched for genes with increased expression in cluster 2. GO categories for genes differentially expressed between PLS clusters. Categories enriched for genes with increased expression in cluster 2 are shown. Numbers of significant and expected genes shown, together with *p* values (Fisher’s exact test). **Table S9.** GO categories enriched for genes with increased expression in cluster 1. GO categories for genes differentially expressed between PLS clusters. Categories enriched for genes with increased expression in cluster 1 are shown. Numbers of significant and expected genes shown, together with *p* values (Fisher’s exact test). (XLSX 4875 kb) Additional file 4: Figure S3. Network analysis of HSF1 and relationship with observed differentially expressed genes in heat shock response. We constructed a network between HSF1 and observed differentially expressed genes following heat shock (1.2 FC, FDR <0.01) in LCLs using IPA, with *lines* denoting the shortest path between HSF1 and a particular molecule or the shortest path plus one molecule. *Radial layout* with the names of molecules showing multiple (nodal) relationships *highlighted*. Other individual molecules also shown. (PDF 546 kb)

## Abbreviations

ChIP-seq: Chromatin immunoprecipitation analysed by high throughput sequencing
EBV: Epstein-Barr virus
eQTL: Expression quantitative trait locus
eSNP: Expression-associated SNP
FC: Fold change
FDR: False discovery rate
GO: Gene ontology
GWAS: Genome-wide association study
HSE: Heat shock element
HSF1: Heat shock factor 1
HSF2: Heat shock factor 2
IPA: Ingenuity Pathway Analysis
LD: Linkage disequilibrium
PLS: Partial least squares
QC: Quality control
QTL: Quantitative trait locus
SNP: Simple nucleotide polymorphism
YRI: Yoruba from Ibadan, Nigeria

## References

[1] Ritossa F (1962). A new puffing pattern induced by temperature shock and DNP in Drosophila. Experientia..

[2] Velichko AK, Markova EN, Petrova NV, Razin SV, Kantidze OL (2013). Mechanisms of heat shock response in mammals. Cell Mol Life Sci..

[3] Gasch AP, Spellman PT, Kao CM, Carmel-Harel O, Eisen MB, Storz G (2000). Genomic expression programs in the response of yeast cells to environmental changes. Mol Biol Cell..

[4] Richter K, Haslbeck M, Buchner J (2010). The heat shock response: life on the verge of death. Mol Cell..

[5] Matsuura H, Ishibashi Y, Shinmyo A, Kanaya S, Kato K (2010). Genome-wide analyses of early translational responses to elevated temperature and high salinity in Arabidopsis thaliana. Plant Cell Physiol..

[6] Richmond CS, Glasner JD, Mau R, Jin H, Blattner FR (1999). Genome-wide expression profiling in Escherichia coli K-12. Nucleic Acids Res..

[7] Rohlin L, Trent JD, Salmon K, Kim U, Gunsalus RP, Liao JC (2005). Heat shock response of Archaeoglobus fulgidus. J Bacteriol..

[8] Tabuchi Y, Takasaki I, Wada S, Zhao QL, Hori T, Nomura T (2008). Genes and genetic networks responsive to mild hyperthermia in human lymphoma U937 cells. Int J Hyperthermia..

[9] Murray JI, Whitfield ML, Trinklein ND, Myers RM, Brown PO, Botstein D (2004). Diverse and specific gene expression responses to stresses in cultured human cells. Mol Biol Cell..

[10] Jego G, Hazoume A, Seigneuric R, Garrido C (2013). Targeting heat shock proteins in cancer. Cancer Lett..

[11] Merkling SH, Overheul GJ, van Mierlo JT, Arends D, Gilissen C, van Rij RP (2015). The heat shock response restricts virus infection in Drosophila. Sci Rep..

[12] Murshid A, Eguchi T, Calderwood SK (2013). Stress proteins in aging and life span. Int J Hyperthermia..

[13] Zugel U, Kaufmann SH (1999). Role of heat shock proteins in protection from and pathogenesis of infectious diseases. Clin Microbiol Rev..

[14] Gibson G, Powell JE, Marigorta UM (2015). Expression quantitative trait locus analysis for translational medicine. Genome Med..

[15] Fairfax BP, Knight JC (2014). Genetics of gene expression in immunity to infection. Curr Opin Immunol..

[16] Fairfax BP, Humburg P, Makino S, Naranbhai V, Wong D, Lau E (2014). Innate immune activity conditions the effect of regulatory variants upon monocyte gene expression. Science..

[17] Davenport EE, Burnham KL, Radhakrishnan J, Humburg P, Hutton P, Mills TC (2016). Genomic landscape of the individual host response and outcomes in sepsis: a prospective cohort study. Lancet Respir Med..

[18] Ye CJ, Feng T, Kwon HK, Raj T, Wilson MT, Asinovski N (2014). Intersection of population variation and autoimmunity genetics in human T cell activation. Science..

[19] Caliskan M, Baker SW, Gilad Y, Ober C (2015). Host genetic variation influences gene expression response to rhinovirus infection. PLoS Genet..

[20] Lee MN, Ye C, Villani AC, Raj T, Li W, Eisenhaure TM (2014). Common genetic variants modulate pathogen-sensing responses in human dendritic cells. Science..

[21] Norry FM, Larsen PF, Liu Y, Loeschcke V (2009). Combined expression patterns of QTL-linked candidate genes best predict thermotolerance in Drosophila melanogaster. J Insect Physiol..

[22] Sambucetti P, Scannapieco AC, Loeschcke V, Norry FM (2013). Heat-stress survival in the pre-adult stage of the life cycle in an intercontinental set of recombinant inbred lines of Drosophila melanogaster. J Exp Biol..

[23] Vieira C, Pasyukova EG, Zeng ZB, Hackett JB, Lyman RF, Mackay TF (2000). Genotype-environment interaction for quantitative trait loci affecting life span in Drosophila melanogaster. Genetics..

[24] Rodriguez M, Snoek LB, Riksen JA, Bevers RP, Kammenga JE (2012). Genetic variation for stress-response hormesis in C. elegans lifespan. Exp Gerontol.

[25] Quinn NL, McGowan CR, Cooper GA, Koop BF, Davidson WS (2011). Identification of genes associated with heat tolerance in Arctic charr exposed to acute thermal stress. Physiol Genomics..

[26] Dixon AL, Liang L, Moffatt MF, Chen W, Heath S, Wong KC (2007). A genome-wide association study of global gene expression. Nat Genet..

[27] Maugeri N, Radhakrishnan J, Knight JC (2010). Genetic determinants of HSP70 gene expression following heat shock. Hum Mol Genet..

[28] International HapMap Consortium (2005). A haplotype map of the human genome. Nature.

[29] International HapMap Consortium (2004). Integrating ethics and science in the International HapMap Project. Nat Rev Genet.

[30] Barbosa-Morais NL, Dunning MJ, Samarajiwa SA, Darot JF, Ritchie ME, Lynch AG (2010). A re-annotation pipeline for Illumina BeadArrays: improving the interpretation of gene expression data. Nucleic Acids Res..

[31] Huber W, von Heydebreck A, Sultmann H, Poustka A, Vingron M (2002). Variance stabilization applied to microarray data calibration and to the quantification of differential expression. Bioinformatics..

[32] Johnson WE, Li C, Rabinovic A (2007). Adjusting batch effects in microarray expression data using empirical Bayes methods. Biostatistics..

[33] Ritchie ME, Phipson B, Wu D, Hu Y, Law CW, Shi W (2015). limma powers differential expression analyses for RNA-sequencing and microarray studies. Nucleic Acids Res.

[34] Carlson M. TxDb.Hsapiens.UCSC.hg19.knownGene: Annotation Package for TxDb Object(s). R package version 3.1.2. 2015.

[35] Alexa A, Rahnenfuhrer J. TopGO: TopGO: Enrichment Analysis for Gene Ontology. R package version 2.20.0. 2010.

[36] Vihervaara A, Sergelius C, Vasara J, Blom MA, Elsing AN, Roos-Mattjus P (2013). Transcriptional response to stress in the dynamic chromatin environment of cycling and mitotic cells. Proc Natl Acad Sci U S A..

[37] Pachkov M, Erb I, Molina N, van Nimwegen E (2007). SwissRegulon: a database of genome-wide annotations of regulatory sites. Nucleic Acids Res..

[38] Stojnic R, Diez D. PWMEnrich: PWM Enrichment Analysis. R package version 4.4.0. 2014.

[39] International HapMap C (2003). The International HapMap Project. Nature.

[40] Chang CC, Chow CC, Tellier LC, Vattikuti S, Purcell SM, Lee JJ (2015). Second-generation PLINK: rising to the challenge of larger and richer datasets. Gigascience..

[41] O’Reilly PF, Hoggart CJ, Pomyen Y, Calboli FC, Elliott P, Jarvelin MR (2012). MultiPhen: joint model of multiple phenotypes can increase discovery in GWAS. PLoS One..

[42] Cusanovich DA, Pavlovic B, Pritchard JK, Gilad Y (2014). The functional consequences of variation in transcription factor binding. PLoS Genet..

[43] Wong D, Lee W, Humburg P, Fairfax BP, Lau E, Chan K (2014). Genomic mapping of the MHC transactivator CIITA using an integrated ChIp-seq and genetical genomics approach. Genome Res..

[44] Pages H. SNPlocs.Hsapiens.dbSNP142.GRCh37: SNP Locations for Homo Sapiens (DbSNP Build 142). R package version 0.99.5. 2014.

[45] Shabalin AA (2012). Matrix eQTL: ultra fast eQTL analysis via large matrix operations. Bioinformatics..

[46] Biswas S, Storey JD, Akey JM (2008). Mapping gene expression quantitative trait loci by singular value decomposition and independent component analysis. BMC Bioinformatics..

[47] Fehrmann RS, Jansen RC, Veldink JH, Westra HJ, Arends D, Bonder MJ (2011). Trans-eQTLs reveal that independent genetic variants associated with a complex phenotype converge on intermediate genes, with a major role for the HLA. PLoS Genet..

[48] Westra HJ, Peters MJ, Esko T, Yaghootkar H, Schurmann C, Kettunen J (2013). Systematic identification of trans eQTLs as putative drivers of known disease associations. Nat Genet..

[49] Pettersson FH, Berglund A (2005). Interpretation and validation of PLS models for microarray data. Chemometrics Chemoinformatics..

[50] Johansson D, Lindgren P, Berglund A (2003). A multivariate approach applied to microarray data for identification of genes with cell cycle-coupled transcription. Bioinformatics..

[51] Mohr B. Genomic mapping of determinants of the transcriptional response to hypoxia in human lymphoblastoid cell lines. DPhil thesis. Oxford: Oxford University; 2010.

[52] Liu Y, Beyer A, Aebersold R (2016). On the dependency of cellular protein levels on mRNA abundance. Cell..

[53] Ryno LM, Genereux JC, Naito T, Morimoto RI, Powers ET, Shoulders MD (2014). Characterizing the altered cellular proteome induced by the stress-independent activation of heat shock factor 1. ACS Chem Biol..

[54] Zhang C, Saunders AJ (2009). An emerging role for Ubiquilin 1 in regulating protein quality control system and in disease pathogenesis. Discov Med..

[55] Bertram L, Hiltunen M, Parkinson M, Ingelsson M, Lange C, Ramasamy K (2005). Family-based association between Alzheimer’s disease and variants in UBQLN1. N Engl J Med..

