# Supplementary figures and images for: Characterisation of the global transcriptional response to heat shock and the impact of individual genetic variation
Source: Genome Med. 2016 Aug 24;8(1):87. doi: 10.1186/s13073-016-0345-5 (PMC4995779; doi:10.1186/s13073-016-0345-5)

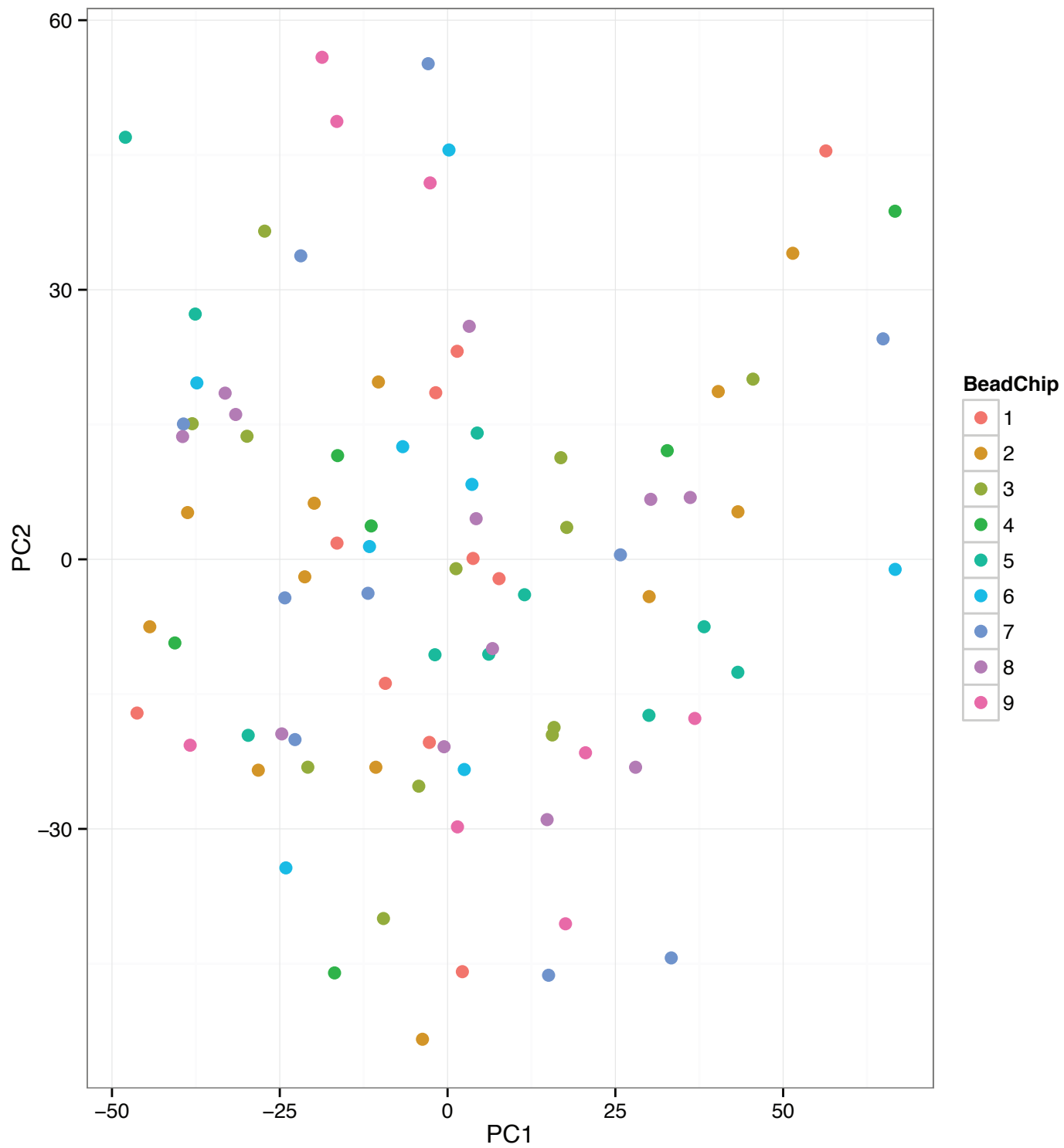

**Figure S1.** PCA plot of ComBat corrected gene expression.

Supplement: Additional file 1: Figure S1. — PCA plot of ComBat corrected gene expression. PCA plot for gene expression in LCLs following heat shock post microarray processing and QC with individual lines coloured by BeadChIP. (PDF 166 kb) [file 13073_2016_345_MOESM1_ESM.pdf]

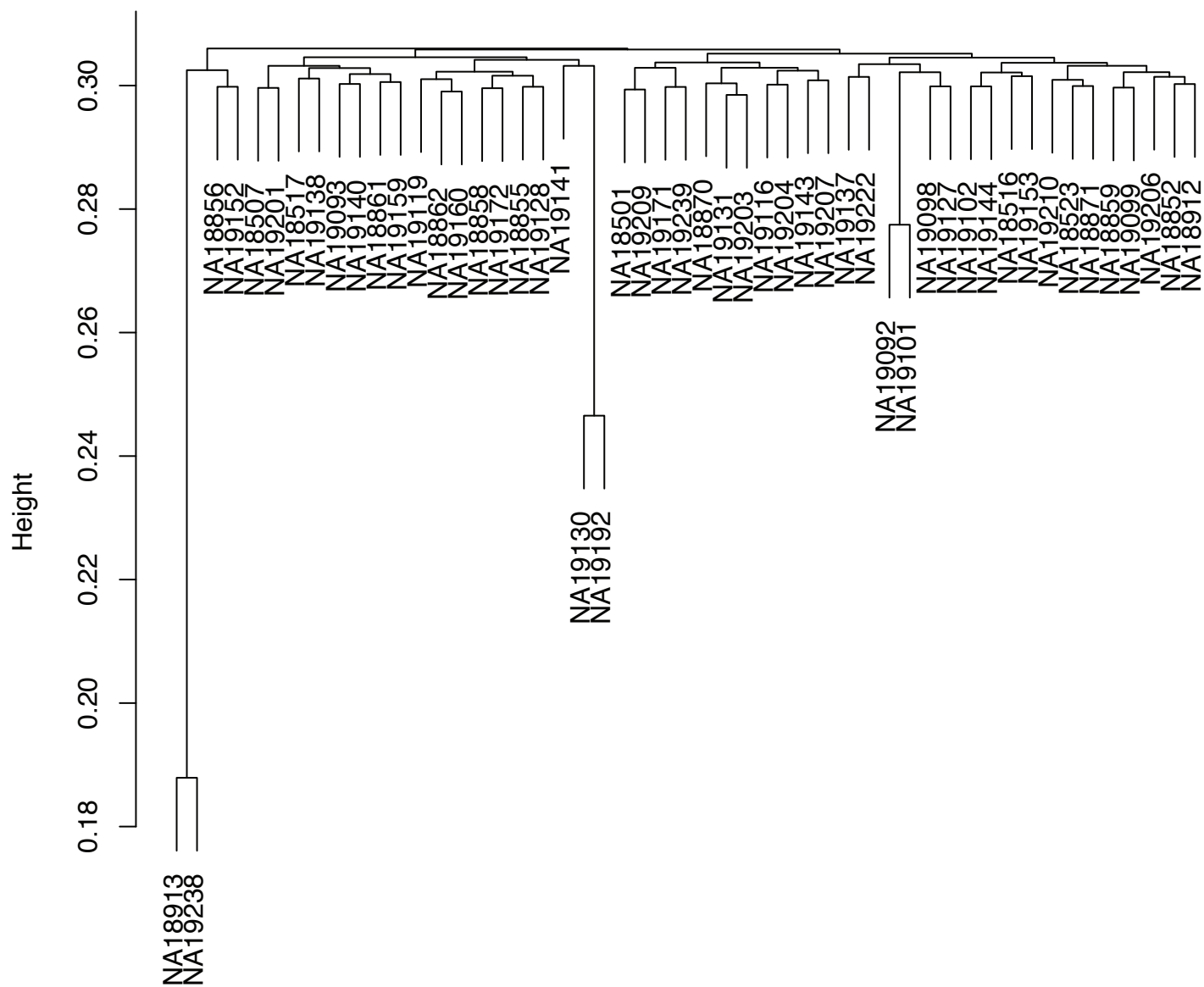

IBS

**Figure S2.** Dendrogram of individuals included in study.

Supplement: Additional file 2: Figure S2. — Dendrogram of individuals included in study. Plot showing distances based on identity by state for LCLs from HapMap (YRI) included in this study. Three pairs show clear indications of increased relatedness. (PDF 131 kb) [file 13073_2016_345_MOESM2_ESM.pdf]
