# Supplementary material for: Characterisation of the global transcriptional response to heat shock and the impact of individual genetic variation
Source: Genome Med. 2016 Aug 24;8(1):87. doi: 10.1186/s13073-016-0345-5 (PMC4995779; doi:10.1186/s13073-016-0345-5)
Supplement: Additional file 4: Figure S3. — Network analysis of HSF1 and relationship with observed differentially expressed genes in heat shock response. We constructed a network between HSF1 and observed differentially expressed genes following heat shock (1.2 FC, FDR <0.01) in LCLs using IPA, with lines denoting the shortest path between HSF1 and a particular molecule or the shortest path plus one molecule. Radial layout with the names of molecules showing multiple (nodal) relationships highlighted. Other individual molecules also shown. (PDF 546 kb) [file 13073_2016_345_MOESM4_ESM.pdf]

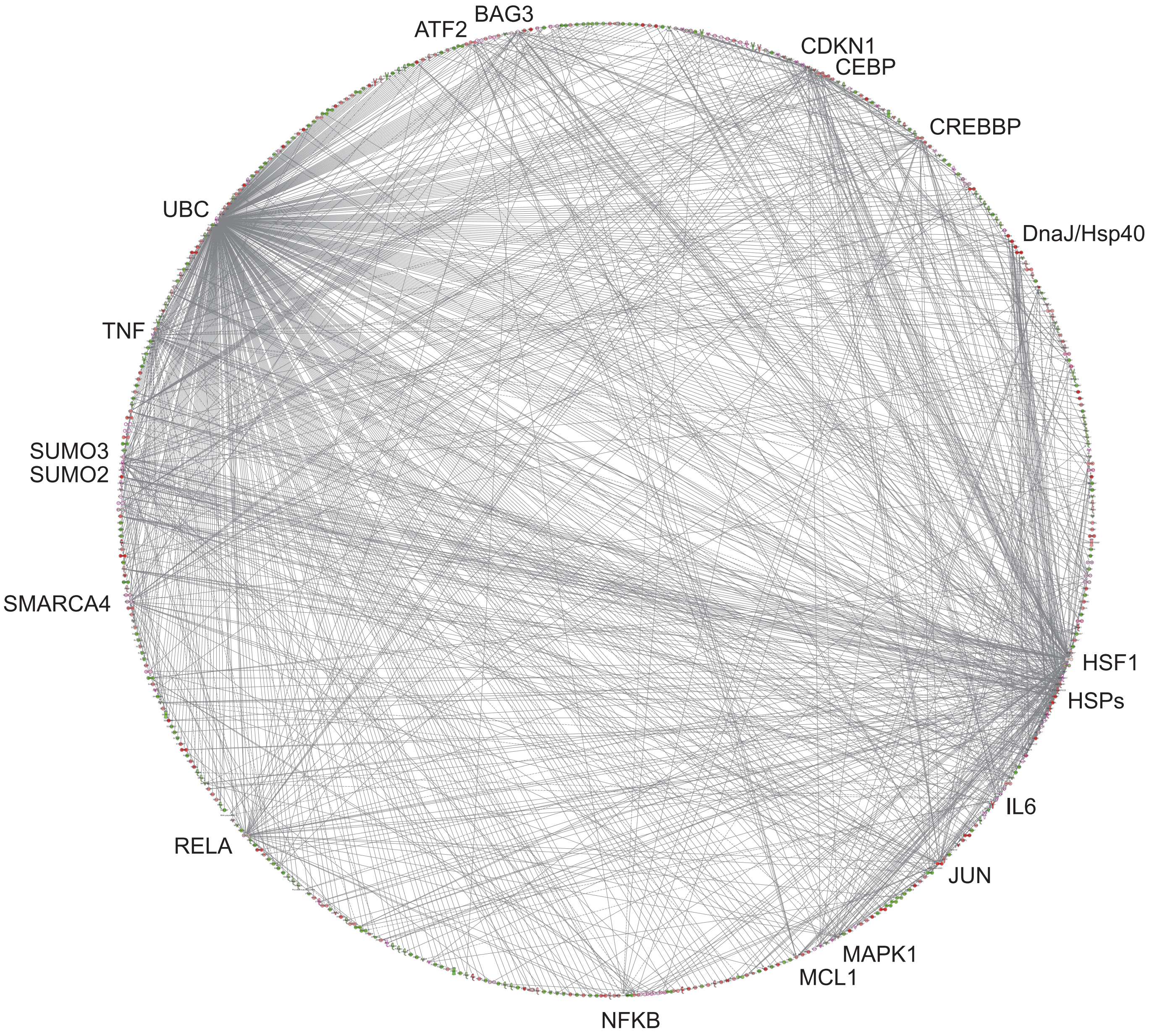

Figure S3. Network analysis of HSF1 and relationship with observed differentially expressed genes in heat shock response
